# HLA Class II Presentation Is Specifically Altered at Elevated Temperatures in the B-Lymphoblastic Cell Line JY

**DOI:** 10.1016/j.mcpro.2021.100089

**Published:** 2021-04-29

**Authors:** Laura C. Demmers, Wei Wu, Albert J.R. Heck

**Affiliations:** 1 Biomolecular Mass Spectrometry and Proteomics, Bijvoet Center for Biomolecular Research and Utrecht Institute for Pharmaceutical Sciences, Utrecht University, Utrecht, Netherlands; 2 Netherlands Proteomics Centre, Utrecht, Netherlands

**Keywords:** immunoproteomics, immunopeptidomics, HLA peptide processing, HLA class I peptide presentation, HLA class II peptide presentation, invariant chain, CLIP peptide cluster, temperature-induced adaptations, ER, endoplasmic reticulum, FDR, false discovery rate, HLA, human leukocyte antigen

## Abstract

Human leukocyte antigen (HLA) molecules play critical roles in our adaptive immune system by signaling a cell's health status to the immune system, through presentation of small peptides. Understanding HLA biology is important because of its prominent role in autoimmune diseases and cancer immunotherapy. Although both the HLA class I and class II antigen processing and presentation pathways have been studied extensively, the fundamental rules in HLA class II antigen presentation still remain less understood. To clarify the mechanistic and adaptive differences between the HLA systems, we challenged a B lymphoblastic cell line (JY), widely used as model system in studying antigen presentation, with a high temperature treatment to mimic a “fever-like state”, representing one of the most common physiological responses to infection. In the absence of real invading pathogenic peptides to present, we could focus on delineating the intrinsic HLA pathway adaptations in response to high temperature in this particular cell line. Following a three-pronged approach, we performed quantitative analyses of the proteome, the HLA class I ligandome, as well as the HLA class II ligandome. The data reveals that elevated temperature may already prepare these cells for an immune-like response through increased HLA class II presentation capacity and specific release of, from the invariant chain originating, CLIP peptides. Interestingly, at high temperature, prominent changes in the composition of the CLIP repertoire were observed, with enrichment of peptides containing C-terminal extensions beyond the CLIP-core region. Collectively, these illustrate intriguing temperature sensitive adaptations in this B cell line.

## Highlights

• Growing JY cells at 40 °C induces similar cellular adaptations as in infection.
• Human leukocyte antigen class II proteins are upregulated in JY cells at 40 °C.
• Heat treatment severely affects the HLA class II system in JY cells.
• CLIP peptides are N terminal extended at 37 °C but C terminal extended at 40 °C.

## Graphical Abstract

**Figure:**
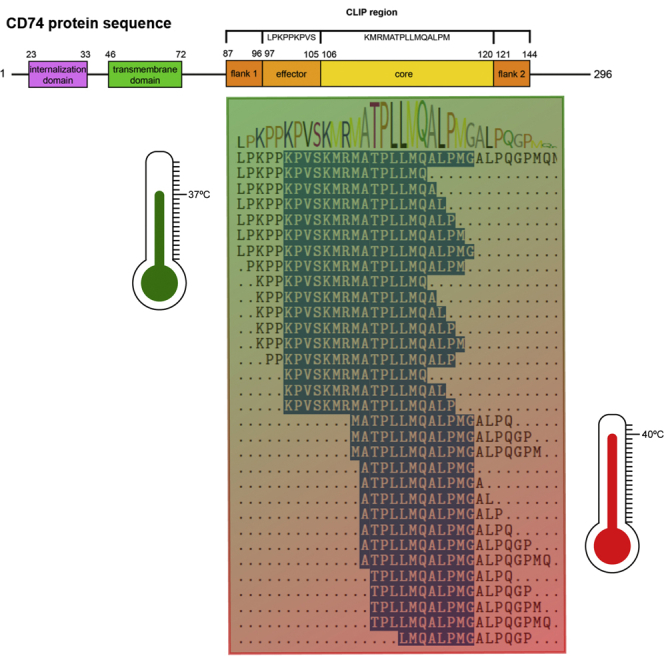

## In Brief

Here the cellular response in a “fever-mimicking state” was investigated by sampling in parallel the proteome and the HLA class I and II ligandomes. Using quantitative proteomics and immunopeptidomics, we found that a proteomic “fever response” is initiated in B-cells after growing the cells for only 3 days at 40 °C and that it is largely mediated by adaptation in the HLA class II rather than HLA class I system.

Human leukocyte antigen (HLA) class I and class II molecules are transmembrane proteins that present small peptide ligands (8- to 12-mers, and 13- to 18-mers respectively) from either endogenous or exogenous proteins to the immune system. Thereby, they play a crucial role in our adaptive immune defense (1). These peptides also reflect the health status of the presenting cells. Usually, the presentation of a “self” peptide can be tolerated by the immune system, whereas cells presenting a “non-self” peptide, such as viral, bacterial, or mutated peptide, will be destroyed when the immune system is triggered. This prevents the further propagation of viral and/or bacterial infections and puts a stop to cancer initiation. Since failure to eliminate such diseased cells can lead to severe consequences, it is critical to fully understand HLA class I and HLA class II peptide ligand processing and presentation.

HLA peptide processing and presentation is increasingly studied in view of its potential therapeutic role in personalized anticancer therapy (2, 3, 4, 5). While similarities exist between the HLA class I and HLA class II presentation pathways, there are also key differences that distinguish these pathways. HLA class I peptide ligands are presented to CD8+ T-cells and predominantly sourced endogenously, from peptides that arise from intracellular protein degradation or from defective ribosomal products (6, 7). Some of these peptides may then be translocated to the endoplasmic reticulum (ER) *via* antigen peptide transporter one and/or two in the ER membrane (6, 8). In the ER, peptide loading on empty HLA class I molecules proceeds *via* a complex containing tapasin, calreticulin, and ERp57 (9) but also higher affinity peptides constantly compete off lower affinity ones. Upon loading of a high-affinity peptide, the glycan on the HLA class I molecule triggers release from the ER toward the Golgi apparatus and subsequently to the plasma membrane to present its peptide (10). Recently, a tapasin analog, TAPBPR, has been reported. Although these proteins are analogs, TAPBPR does not associate with the peptide loading complex, does not have to reside in the ER and is mutually exclusive with tapasin and therefore plays an alternative role in peptide loading (11). The fact that this pathway is only recently discovered highlights that despite intense past investigations, there is still much more to discover about HLA peptide processing and presentation.

The HLA class II presentation pathway is inherently different. HLA class II peptide ligands are presented to CD4+ T-cells and predominantly sampled from exogenous proteins and endogenous proteins produced *via* autophagy in the endosomal pathway (12). HLA class II complexes are assembled in the ER, where, instead of binding of a high-affinity antigenic peptide, a part of CD74 (invariant chain) binds into the peptide binding groove and targets the molecule into the endosomal pathway (13, 14). HLA class II molecules come in contact with antigenic peptides in the MIIC compartment (15), where the invariant chain is cleaved sequentially by legumain (LGMN), cathepsin S, cathepsin L, and cathepsin F, leaving behind a smaller peptide fragment called CLIP. This HLA class II bound CLIP peptide can be exchanged out for a higher affinity antigenic peptide with help of the chaperones HLA-DM and HLA-DO (16, 17). After loading, the HLA class II complexes with peptide ligands are transported to the plasma membrane through vesicular transport where they are stably inserted (18, 19, 20).

While these HLA class I and class II mechanisms have been studied for a long time in normal homeostatic conditions, still very little is known about how specific cellular stress conditions could alter these pathways, similarly or distinctively. Therefore, we induced heat stress on JY B-cells by a 3-day exposure to high temperature to monitor how HLA processing and presentation are affected. Physiologically, this would be akin to a fever state (21), without actual invading pathogens. From this, we discovered that heat-induced cellular stress alone seemed to re-shape the B-cell proteome and prepares B-cells for an immune response, thereby specifically modulating the ultimate step in HLA class II presentation and stimulating release of specific CD74 CLIP peptides.

## Experimental Procedures

### Cell Culture

The B-lymphoblastoid cell line JY (HLA-A∗02:01, HLA-B∗07:02, HLA-C∗07:02, HLA-DPA1∗01:03, HLA-DPB1∗02:01/04:02, HLA-DQA1∗01:03/03:01, HLA-DQB1∗03:02/06:03, HLA-DRA∗01, HLA-DRB1∗04:04/13:01, HLA-DRB4∗04, HLA-DRB5∗02) was cultured in RPMI 1640 medium (+glutamine, Gibco) supplemented with 10% fetal bovine serum, 50 U/ml penicillin, and 50 μg/ml streptomycin in a humidified incubator at 37 °C with 5% CO_2_. Three days before harvest, the cells were split and grown at either 37 °C or 40 °C in a humidified incubator with 5% CO_2_.

### Proteomics

JY cells were lysed in 8 M urea in 50 mM ammonium bicarbonate supplemented with 1× complete EDTA-free protease inhibitor cocktail (Roche Diagnostics), 50 μg/ml DNAse I (Sigma-Aldrich), and 50 μg/ml RNAse A (Sigma-Aldrich). The lysate was cleared by centrifugation for 1 h at 18,000*g* at 15 °C. The protein concentration was determined with the Bradford assay (Bio-Rad). For each sample, 50 μg of whole cell lysate was reduced, alkylated and digested sequentially with Lys-C (1:100) and trypsin (1:75). The digested peptides were acidified to 0.1% formic acid and purified by SepPak C18 columns (Supelco). Peptide elution was performed with 80% acetonitrile in 0.1% formic acid. The samples were dried by vacuum centrifugation and reconstituted in 2% formic acid before LC-MS/MS analysis. Per sample, three technical replicates were measured by LC-MS/MS.

### HLA Class I and HLA Class II Ligandomics

Per condition, 5 × 10^8^ cells were harvested by centrifugation and washed three times with phosphate buffered saline which had been incubated at the respective growth temperatures. The pelleted cells were disrupted in 10 ml lysis buffer per gram cell pellet for 1.5 h at 4 °C, on gentle end-to-end rotation. The lysis buffer consisted of Pierce IP lysis buffer (Thermo Fischer Scientific) supplemented with 1× complete protease inhibitor cocktail (Roche Diagnostics), 50 μg/ml DNAse I (Sigma-Aldrich), and 50 μg/ml RNAse A (Sigma-Aldrich). The lysate was then cleared by centrifugation for 1 h at 18,000*g* at 4 °C. The protein concentration of the supernatant was determined with the BCA assay (Pierce).

HLA class I immunoaffinity purification was performed as previously described (22). Briefly, HLA class I complexes were purified from 25 mg of lysate, using 0.5 mg W6/32 antibody (23) coupled to 125 μl protein A/G beads (Santa Cruz). To prevent co-elution, the antibodies were cross-linked to protein A/G beads. For retrieval of HLA class II complexes, we used an HLA-DR specific antibody (B8-11-2, Bioceros/Polpharma Biologics) and performed the pulldown using the HLA class I-depleted lysate as input. For both immunoaffinity purifications, incubation took place at 4 °C for approximately 16 h. After immunoaffinity purification, the beads were washed with 40 ml of cold phosphate buffered saline. HLA class I and HLA class II complexes and peptide ligands were eluted with 10% acetic acid. The peptide ligands were separated from the HLA molecules using 10 kDa molecular weight cutoff filters (Millipore) for HLA class I and 30 kDa molecular weight cutoff filters (Millipore) for HLA class II. The flowthrough containing the HLA class I or HLA class II peptide ligands was freeze-dried, reconstituted in 0.1% formic acid for further cleanup by C18 STAGE tips (Thermo Fischer Scientific) and eluted from C18 STAGE tips with 80% acetonitrile, 0.1% formic acid. The samples were dried by vacuum centrifugation and reconstituted in 2% formic acid before LC/MS-MS analysis. Per sample, three technical replicates were measured by LC-MS/MS. The 10 kDa and 30 kDa retentate containing HLA class I and HLA class II proteins were resuspended in 8 M urea and retained for gel analyses.

### Proteome LC-MS/MS Analysis

The data were acquired with an UHPLC 1290 system (Agilent) coupled to a Q-Exactive HFX mass spectrometer (Thermo Fischer Scientific). The peptides were trapped (Dr Maisch Reprosil C18, 3 μM, 2 cm × 100 μM) for 5 min in solvent A (0.1% formic acid in water) before being separated on an analytical column (Agilent Poroshell, EC-C18, 2.7 μM, 50 cm × 75 μM). Solvent B consisted of 80% acetonitrile in 0.1% formic acid. The gradient was as follows: 5 min trapping, followed by 155 min gradient from 10% to 36% solvent B. Subsequently, 10 min of washing with 100% solvent B and 10 min re-equilibration with 100% solvent A. The mass spectrometer operated in data-dependent mode. Full scan MS spectra from m/z 375 to 1600 were acquired at a resolution of 60,000 to a target value of 3 × 10^6^ or a maximum injection time of 20 ms. MS/MS spectra were acquired at a resolution of 15,000. The top 15 most intense precursors with a charge state of two to five were chosen for fragmentation. The HCD fragmentation was performed at 27% normalized collision energy on selected precursors with 16 s dynamic exclusion at a 1.4 m/z isolation window after accumulation to 1 × 10^6^ ions or a maximum injection time of 50 ms.

### Ligandome LC-MS/MS Analysis

The data were acquired with an UHPLC 1290 system (Agilent) coupled to an Orbitrap Fusion Lumos Tribrid mass spectrometer (Thermo Fischer Scientific). Peptides were trapped (Dr Maisch Reprosil C18, 3 μM, 2 cm × 100 μM) for 5 min in solvent A (0.1% formic acid in water) before being separated on an analytical column (Agilent Poroshell, EC-C18, 2.7 μm, 50 cm × 75 μm). Solvent B consisted of 80% acetonitrile in 0.1% formic acid. The gradient was as follows: first 5 min of trapping, followed by 90 min gradient from 7% to 35% solvent B. Subsequently, 10 min of washing with 100% solvent B and 10 min re-equilibration with 100% solvent A. The mass spectrometer operated in data-dependent mode. Full scan MS spectra from m/z 400 to 650 (HLA class I) or m/z 300 to 1500 (HLA class II) were acquired at a resolution of 60,000 after accumulation to a target value of 4 × 10^5^ or a maximum injection time of 50 ms (HLA class I) or 250 ms (HLA class II). MS/MS spectra were acquired at a resolution of 15,000. Up to three most intense precursors with a charge state of two or three starting at m/z 100 (HLA class I) or charge state two to five (HLA class II) were chosen for fragmentation. For peptide identification, EThcD fragmentation (24) was performed at 35% normalized collision energy on selected precursors with 18 s dynamic exclusion (HLA class I) or 60 s dynamic exclusion (HLA class II) after accumulation of 5 × 10^4^ ions or a maximum injection time of 250 ms (HLA class I) or 1500 ms (HLA class II).

### Proteome Data Analysis

Raw files were searched using MaxQuant, version 1.6.10.0, and the Andromeda search engine against the human Uniprot database (20,431 entries, downloaded in December 2019) edited with the JY-specific HLA proteins. Enzyme specificity was set to trypsin and up to two missed cleavages were allowed. Cysteine carbamidomethylation was set as fixed modification. Methionine oxidation and N-terminal acetylation were set as variable modifications. Precursor mass tolerance was set to 20 ppm. Fragment ion tolerance was set to 4.5 ppm. False discovery rate (FDR) was restricted to 1% in both protein and peptide quantification. For quantitative comparisons, label-free quantification (based on unique + razor peptides) was enabled with “match between runs”. For HLA protein quantification, the label-free quantification was based on unique peptides only. Data normalization and statistics were performed with Perseus, version 1.6.7.0. Gene ontology analysis was performed with Database for Annotation, Visualization, and Integrated Discovery, version 6.8 (DAVID (25)). The data were visualized with Graphpad Prism 8.0.

### Ligandome Data Analysis

Raw files were searched using Sequest HT in Proteome Discoverer 2.2 against the Swiss-Prot human database (20,258 entries, downloaded in February 2018) edited with JY-specific HLA proteins and 20 most abundant FBS contaminants (26). The search was set to unspecific with a minimum precursor mass of 797 Da to a maximum precursor mass of 1950 Da (HLA class I) or a minimum precursor mass of 350 Da to a maximum precursor mass of 5000 Da (HLA class II) with a mass tolerance of 10 ppm. Fragment ion tolerance was set to 0.02 Da. The identified peptides were filtered against 1% FDR using the Percolator algorithm, 5% peptide FDR and Xcorr >1. Cysteine cysteinylation and methionine oxidation were set as variable modifications. From the identified peptides, FBS contaminants were removed. Binding affinity of HLA class I peptide ligands was predicted with NetMHCpan-4.0 (27) with a binder cutoff at rank 2. Binding affinity of HLA class II ligands was predicted with NetMHCIIpan-4.0 (28) with a binder cutoff of <1000 nM. Alignments were made using the msa R package. The data were visualized with Graphpad Prism 8.0.

### Experimental Design and Statistical Rationale

For each proteome and HLA peptidome biological sample, three technical replicates were measured. These samples were injected from separate injection wells to prevent evaporation and thereby concentration of the samples. Proteome LFQ intensities were extracted by MaxQuant and were Log2 transformed in Perseus. The proteome identifications were filtered for at least two valid values in at least one of the conditions. Missing values were imputed based on a normal distribution. Pairwise comparisons were performed using a student's *t* test (two-sided, adjusted *p*-value < 0.05).

## Results

### Composition of JY HLA Class I and Class II Ligandomes

**Fig. 1:**
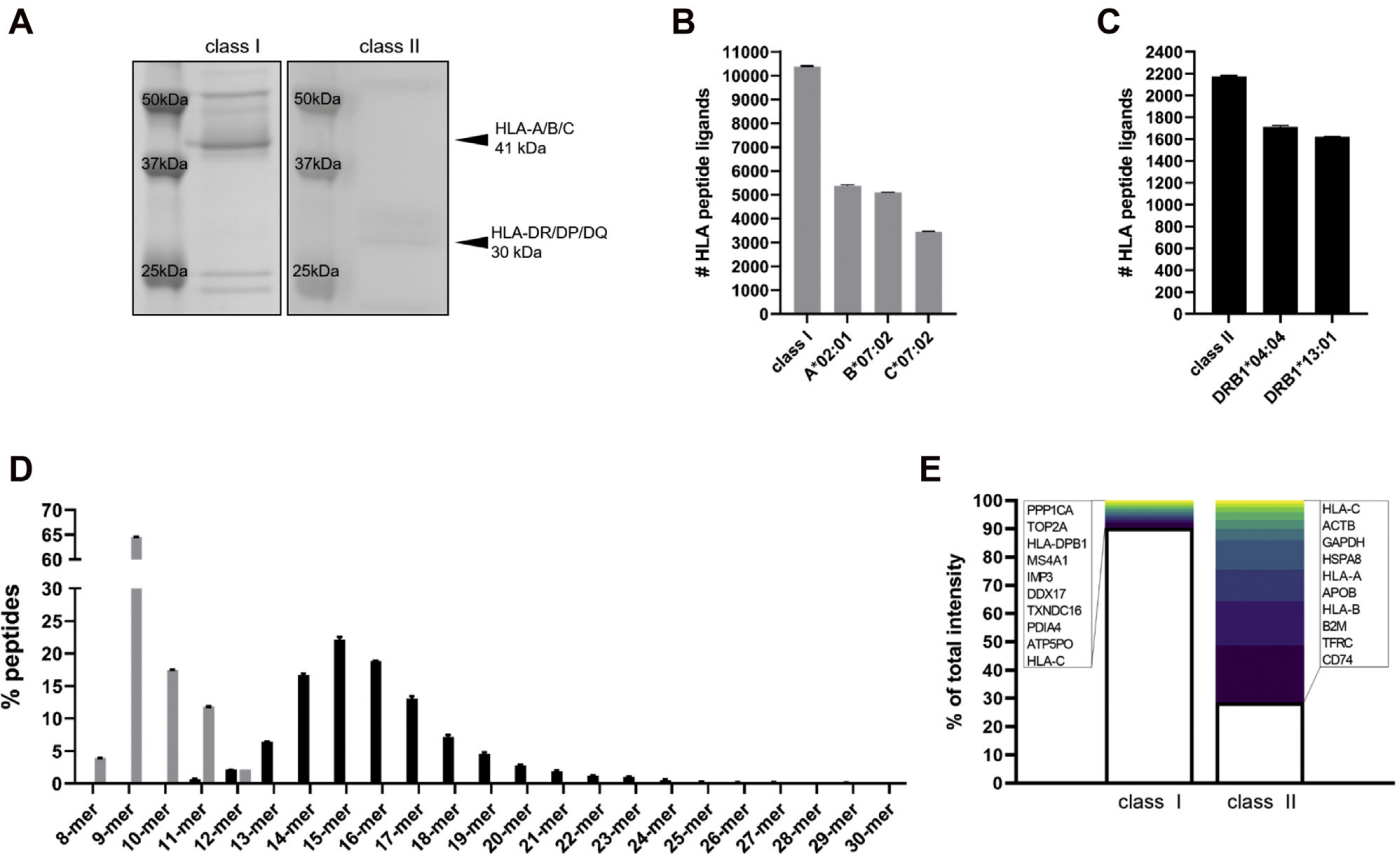
**HLA class I and class II peptide ligand characteristics.***A*, Coomassie-stained gel of immunoprecipitated HLA class I and HLA class II proteins. The HLA class I and HLA class II bands are visible at 41 kDa and 30 kDa respectively. *B*, total number of HLA class I peptide ligands identified. First bar shows the total number, the other bars the distribution per HLA type. *C*, total number of HLA class II peptide ligands identified and their distribution over the different HLA types. *D*, HLA class I (*gray*) and HLA class II (*black*) peptide ligand length distributions. *E*, abundance based top ten source proteins contributing to the HLA class I and HLA class II peptide ligandomes. HLA, human leukocyte antigen.

To investigate how the B-cell proteome and ligandome changes upon cellular stress, JY cells were grown at 37 °C and subsequently split into two and further grown for 3 days at either 37 °C or at 40 °C before analysis. From these paired materials, we purified HLA class I and HLA class II complexes and peptide ligands sequentially from the JY cell line, grown at 37 °C. The JY cell line is homozygous for HLA-A∗02:01, HLA-B∗07:02, HLA-C∗07:02, which simplifies the interpretation of HLA class I peptide presentation and makes it a widely used benchmarking cell line in immunopeptidomics (22, 29, 30, 31, 32, 33, 34, 35). In terms of HLA class II, JY cells have the alleles HLA-DPA1∗01:03, HLA-DPB1∗02:01/04:02, HLA-DQA1∗01:03/03:01, HLA-DQB1∗03:02/06:03, HLA-DRA∗01, HLA-DRB1∗04:04/13:01, HLA-DRB4∗04, and HLA-DRB5∗02, as verified by HLA typing *via* next generation sequencing. By means of Coomassie staining, we verified the specific immunoaffinity capture of HLA class I and HLA class II proteins on SDS-PAGE at the respective molecular weights (Fig. 1*A*). This gave us confidence that sequential purification of HLA class I and class II complexes from the same lysate was feasible, with a detectable yield.

From HLA class I purification, we identified >10,000 unique peptide ligands (10,380 ± 24, Fig. 1*B*), over three technical replicates. Within this, 93% of all peptides were predicted to bind to HLA-A∗02:01, HLA-B∗07:02, or HLA-C∗07:02. The smaller number of HLA-C binders is likely because of lower protein copy number of HLA-C relative to the other class I HLAs (36, 37). In contrast to the large identification of HLA class I peptide ligands, only about 2100 HLA class II peptides (2173 ± 10, Fig. 1*C*) were identified. This lower identification number, though still with high specificity of 81%, is expected and consistent with the lower expression level of HLA class II on JY cells. Here, we only predicted the binding affinity against HLA-DR alleles, because we performed the HLA class II immunoaffinity purification with a DR-specific antibody, unlike in the HLA class I purification where a pan-HLA class I antibody (W6/32) was used. The combined peptide numbers for HLA-A/B/C and HLA-DRB1 exceed 100% as the binding motifs for the alleles can be similar. In that way, peptides can be assigned to multiple alleles. As expected, HLA class II peptide ligands were also substantially longer with a mode of 15 residues, compared with nine residues for HLA class I (Fig. 1*D*).

Interestingly, HLA class II peptides sampled from the top ten source proteins made up almost 70% in intensity in each measurement, suggesting that fragments of these 10 source proteins heavily dominate the HLA class II peptide ligandome, in the absence of pathogenic peptides to present. Among these was CD74, the invariant chain precursor protein of the CLIP peptide(s), which is known to bind and aid the folding of HLA class II molecules. Peptides originating from class I HLA proteins were also among the top contributors to HLA class II ligandome presentation. This is likely because of the obligatory retrograde recycling of plasma membrane HLA class I molecules during HLA class II presentation (38, 39). In a striking cross comparison, the top ten source proteins only contributed about 10% of the HLA class I peptide ligands (Fig. 1*E*), reflecting fundamental differences in the sampling space of HLA class I and class II peptide presentation.

### The CD74 Peptide Cluster Is the Most Sampled Self-Protein in the HLA Class II Repertoire

**Fig. 2:**
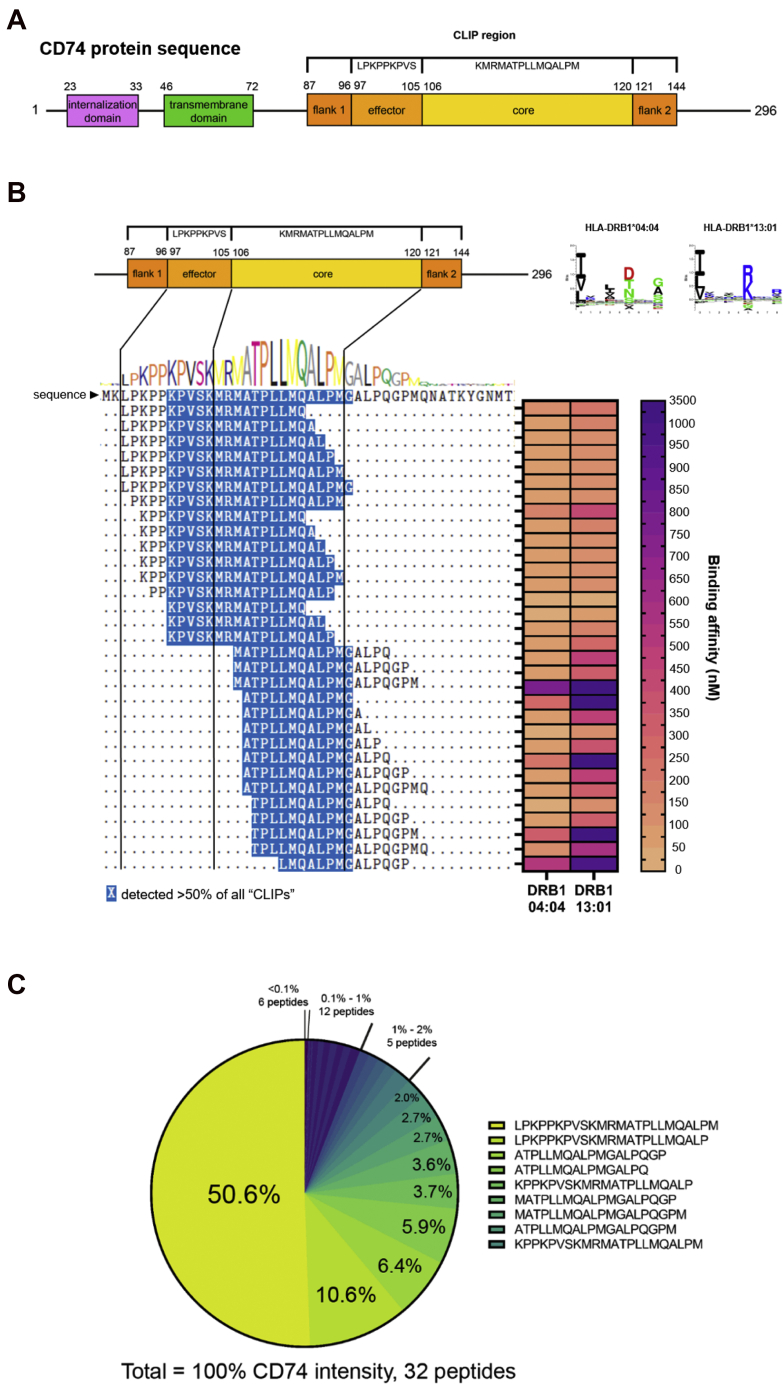
**CLIP is a repertoire of peptides.***A*, domain schematic of the CD74 protein, annotating the internalization, transmembrane and CLIP regions. *B*, alignment of the identified CLIP peptide cluster with their corresponding binding affinity (nM) for HLA-DRB1∗04:04 and HLA-DRB1∗13:01. *C*, pie chart of all identified CLIP peptides with contributing intensity as a fraction of total intensity of peptides originating from the CD74 CLIP region for cells grown at 37 °C. HLA, human leukocyte antigen.

Given the high occupancy of CD74 peptides on HLA class II molecules (~20%, Fig. 1*E*), we examined the sequence features and intensities of all these CD74 peptides more closely. CD74 is a 296 amino acid protein that plays a key role in HLA class II peptide loading. During the early steps of HLA class II peptide loading, the CLIP region of CD74 (Fig. 2*A*) is known to be inserted into the HLA class II binding groove to stabilize the complex and prevent unspecific binding of other peptides, until the molecule has reached the endosomal compartment where the intended peptide cargo is picked up (14, 40, 41, 42). With an open HLA class II peptide groove that can accommodate both N-terminal and C-terminal peptide protrusions (43), the HLA class II binding groove was reported to contain four anchoring positions (P1, P4, P6, and P9), where M107, A110, P112, and M115 from the CLIP core region are supposed to bind (17, 44).

In our JY HLA class II peptide ligandome, a total of 32 different peptides from the CD74 CLIP region were detected (Fig. 2*B*). By aligning these peptides against the CD74 CLIP sequence, we noticed that only M115 (out of the four documented anchor positions) is perfectly conserved in all the CLIP peptides detected, whereas almost half of the peptides (15 out of 32) did not include M107. This affirms that what is classically described as the “CLIP peptide” is not a single sequence but a set of different sequences. Moreover, almost all the peptides from CD74 featured extensions outside the core region; 17 peptides were detected with N-terminal extensions, whereas 15 were C terminally extended. By means of NetMHCIIpan predictions, we further verified that all these 32 peptides detected could bind to DRB1∗04:04/13:01 and that each of these peptides can bind to at least one of the two DRB1 alleles of the JY cell line, with <1000 nM affinity. Despite apparent sequence laddering, the intensity distribution of these 32 CLIP peptides was far from uniform. For instance, a LPKPPKPVSKMRMATPLLMQALPM peptide contributed about 50% in intensity, and eight other peptides together constitute another 38% in intensity (Fig. 2*C*, supplemental Table S1), suggesting that in addition to sequence variation, intensity, which may be used as a proxy for HLA class II groove occupancy, could also differ. Collectively these data consolidate the CLIP peptide repertoire and proportion in normal growth conditions at 37 °C.

### High Temperature Prepares B Cells for an HLA Class II Immune Response

With extensive HLA class I and class II peptide ligandome characterization of the JY cell line and resting characteristics of the CLIP peptide repertoire fully documented, we next challenged the model system with a 3-day heat treatment at 40 °C, to simulate a prolonged fever response though in the absence of real invading pathogens. We then analyzed the total proteome together with the HLA class I and class II peptides for temperature-induced changes along the antigen presentation pathway and consequences on the ligandome. Without the competition for loading and interference of foreign antigens, we aimed to better focus on the intrinsic response and adaptations on antigen presentation.

**Fig. 3:**
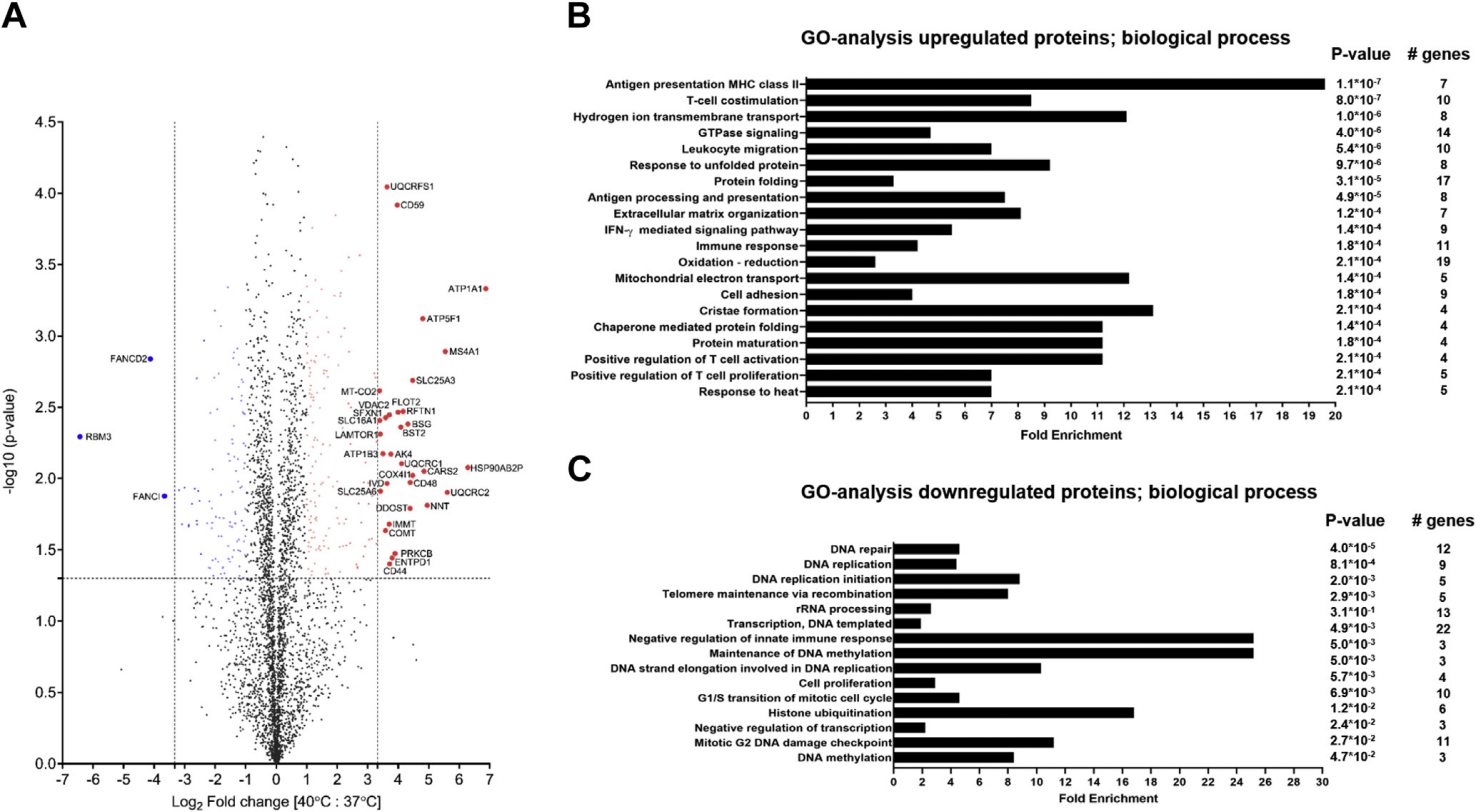
**Proteome alterations at 40 °C.***A*, volcano plot of all changing proteins (n = 3478) at 40 °C *versus* 37 °C. All proteins with a >10-fold increase are indicated in *red* and with a >10-fold decrease in *blue*. *B*, Gene ontology biological process analysis of all upregulated proteins at 40 °C using the full proteome as background. *C*, Gene ontology biological process analysis of all downregulated proteins at 40 °C using the full proteome as background. HLA, human leukocyte antigen.

**Fig. 4:**
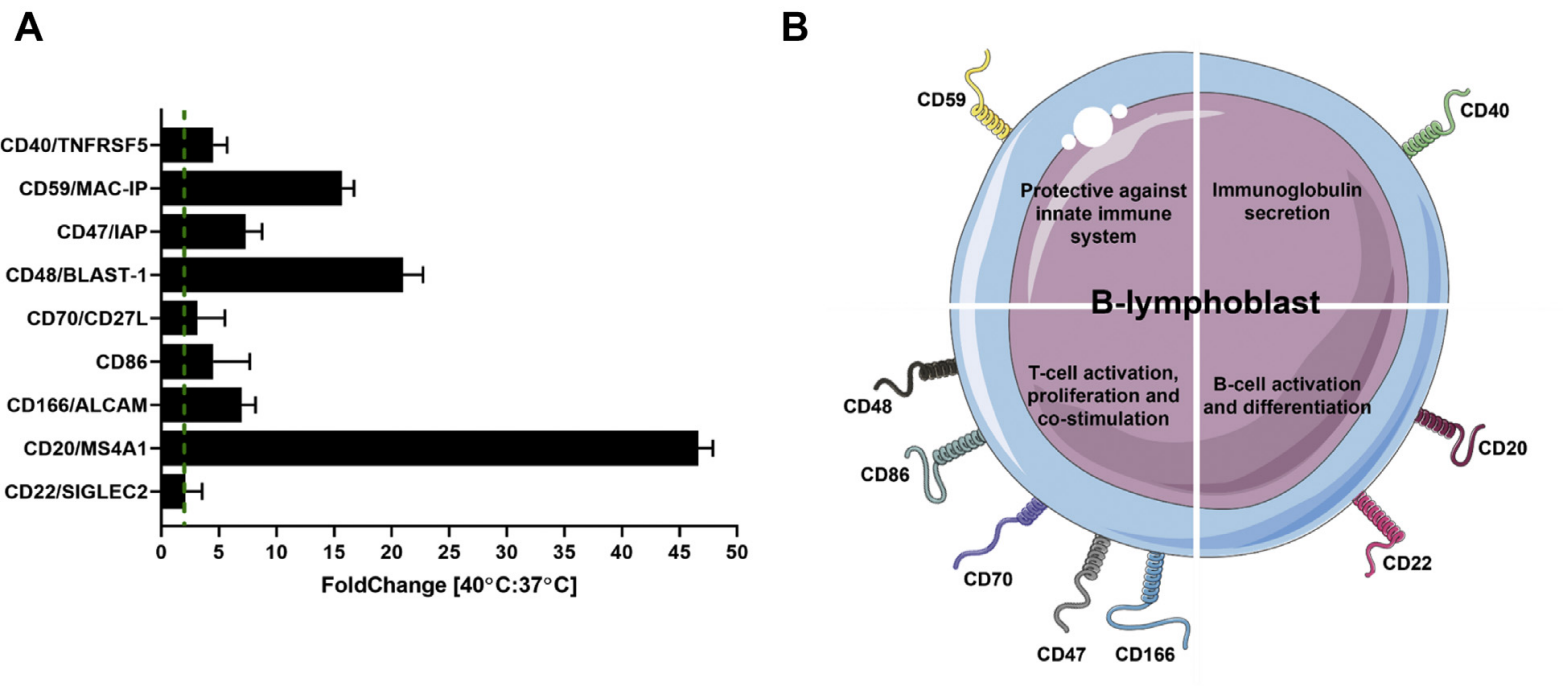
**B-cell preparation at 40 °C.***A*, protein fold change (cutoff at fold change 2) at 40 °C *versus* 37 °C of cell surface cluster of differentiation (CD) proteins. *B*, functional categories of upregulated CD proteins.

As shown in the volcano plot (Fig. 3*A*, supplemental Table S2) summarizing changes in 3478 proteins that were quantifiable in at least two out of three technical replicates, relatively few proteins were regulated by drastic fold-changes, suggesting proteome changes were not widespread but likely quite specific in JY cells subjected to high temperature. Indeed, the proteins that did change in abundance by more than 2-fold (177 upregulated; 103 downregulated) were significantly enriched for functional processes involving regulation of T cell proliferation and activation and interferon-gamma–mediated signaling, both of which are consistent with the biological function of B cells during an infection (45, 46, 47) (Fig. 3, *B* and *C*). Among the upregulated proteins, numerous cell surface localized cluster of differentiation (CD) proteins indicating B cell functional activation were also detected (Fig. 4, *A* and *B*). The presence of CD20 and CD22 has been linked to B-cell activation and differentiation (48, 49, 50), wherease CD86, CD48, CD70, CD47, and CD166 are markers of functional B cells that can engage in T cell co-simulation (51, 52, 53, 54, 55). CD59 is the major protective protein against the membrane attack complex (complement system), ensuring that only invading pathogens are lysed (56), and CD40 is required for immunoglobulin production (57). In the gene ontology enrichment performed on upregulated proteins (Fig. 3*B*), the most significant enrichment was found in the biological process of HLA class II antigen presentation (Fig. 3, *B* and *C*). This affirmed the relevance of heat treatment and our model system to study temperature-induced changes in antigen presentation.

**Fig. 5:**
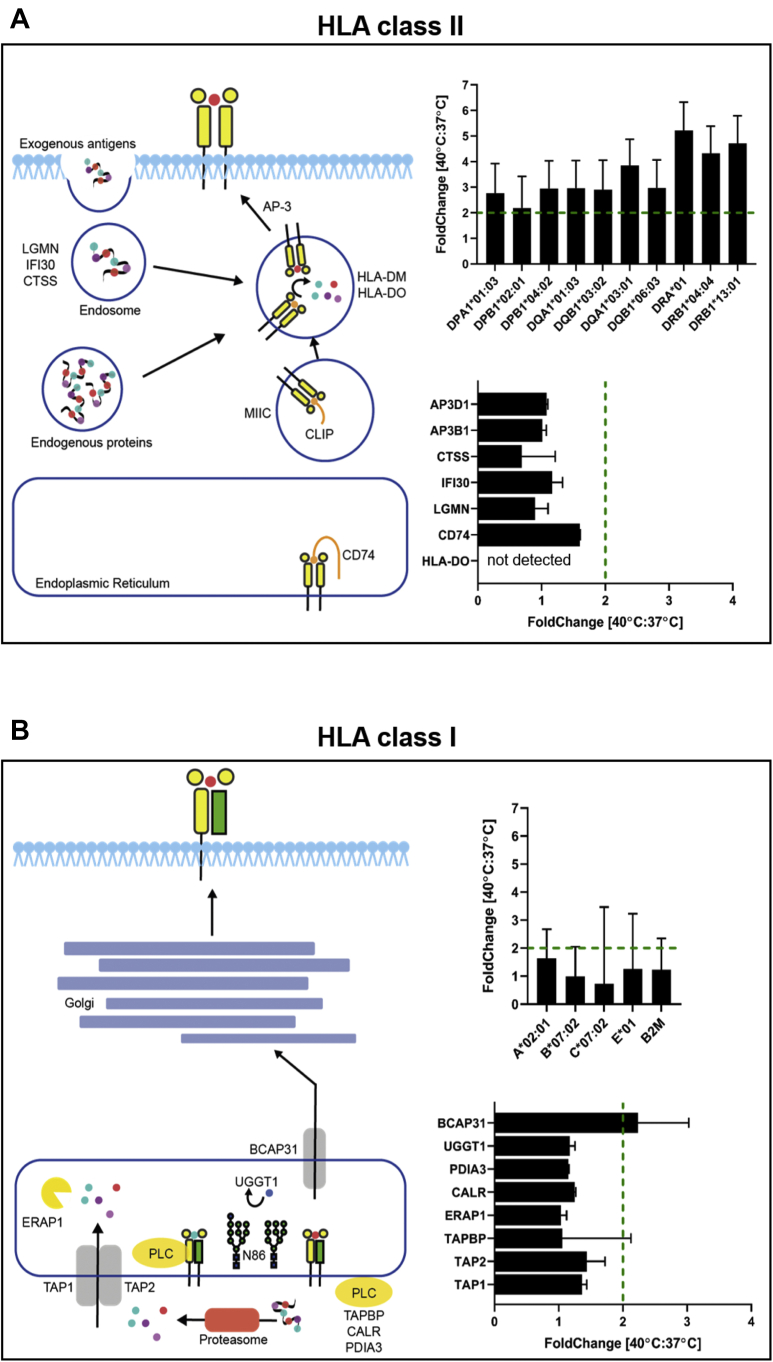
**HLA class II and HLA class I antigen processing and presentation at 40 °C.***A*, schematic of the HLA class II processing and presentation pathways. Annotated are important proteins involved in this process with the respective, substantial, fold change they undergo at elevated temperature (40 °C *versus* 37 °C). *B*, schematic of the HLA class I processing and presentation pathways. Annotated are important proteins involved in this process with the respective, negligible, fold change they undergo at elevated temperature (40 °C *versus* 37 °C). HLA, human leukocyte antigen.

A detailed examination of specific changes in HLA class II antigen processing and presentation pathway revealed that only the abundance of HLA class II molecules seemed to change significantly (>2-fold) on heat treatment, whereas no significant changes were observed elsewhere along the HLA class II antigen presentation route (Fig. 5*A*). A mild elevation in CD74 protein level (of 1.6 fold) might also have correlated additionally with the need to bind more copies of HLA class II proteins en route to the endosome. Collectively, this demonstrates that the adaptations in JY cells, induced by high temperature, likely occur in the final step of HLA class II presentation but not earlier in the capacity to invaginate and breakdown exogenous pathogens (for instance LGMN, IFI30, CTSS, Fig. 5*A*).

In contrast, the HLA class I pathway appeared to be minimally altered at the elevated temperature (Fig. 5*B*), both at the processing and presentation levels. Although a marginal increase in ER transporter BCAP31 was detected, the ER transporter function of BCAP31 is not specific only to the export of loaded HLA class I complexes but also involving general export of many other membrane proteins. This proteome comparison of the presentation machinery thus provides clear evidence that the regulation of HLA class I and class II pathways are controlled distinctly and independently, such that either system may be triggered separately. In addition, the specific upregulation of HLA class II proteins and CD74 appear to be priming steps following high temperature that might have consequences such as boosting HLA class II presentation when pathogens are indeed present in the system.

**Fig. 6:**
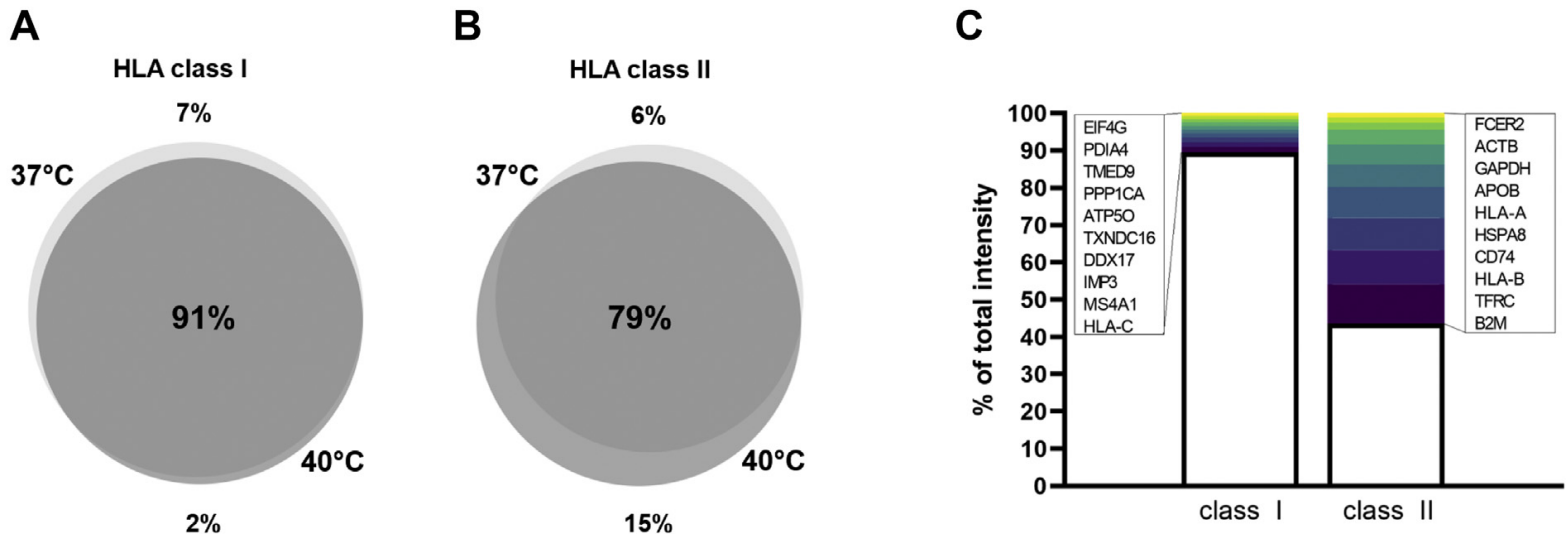
**HLA class I and class II peptide ligand characteristics at 40 °C compared to 37 °C.***A*, overlap of identified HLA class I peptide ligands between 37 °C and 40 °C. *B*, overlap of identified HLA class II peptide ligands between 37 °C and 40 °C. *C*, top ten source proteins of HLA class I and HLA class II peptide ligandomes at 40 °C. HLA, human leukocyte antigen.

**Supplemental Figure S1:**
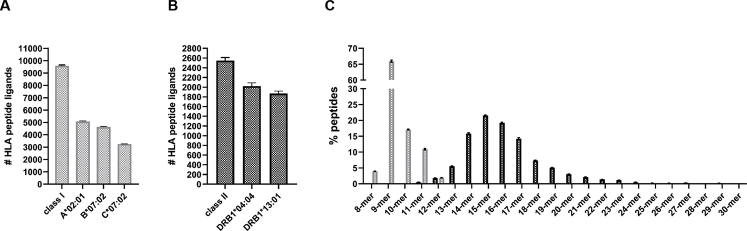
HLA class I and class II peptide ligand characteristics at 40 ºC. A, total number of HLA class I peptide ligands identified and their distribution over the different HLA class I types. B, total number of HLA class II peptide ligands identified and their distribution over the different HLA class II types. C, HLA class I (gray) and HLA class II (black) peptide ligand length distributions. HLA, human leukocyte antigen.

To evaluate this adaptation in high temperature further, at the level of HLA peptides presented, we overlapped either class I or class II peptide ligand species identified at 37 °C and 40 °C (Fig. 6, *A* and *B*). Since the temperature adaptation did not involve increased expression of HLA class I proteins, it was logical that the total number of HLA class I peptide ligands presented did not change significantly (9589 ± 71 peptide ligands) (supplemental Fig. S1), and a large peptide species overlap of 91% was observed. HLA class II peptide ligand species on the other hand were increased by 17% in total (2547 ± 12 peptide ligands) (supplemental Fig. S1). Considering that the abundance of every HLA class II protein increased by more than 100% (Fig. 5*A*), the increase in HLA peptide species seemed low. This implies that at 40 °C, more HLA class II proteins are loaded with seemingly the same repertoire of peptide ligands. This is also evident from Figure 6*C*, where the top ten source proteins contributing peptides to HLA class I and class II presentation remain largely the same as in the case of normal growth at 37 °C (Fig. 1*F*). Specifically, EIF4G and TMED9 (for class I) and FCER2 (for class II) in Figure 6*C* were not among the top ten source proteins at 37 °C (Fig. 1*E*), but all still within the top 40 major source proteins providing peptides for HLA class I and class II loading. Collectively, these data lead us to conclude that at 40 °C, more HLA class II proteins are made, but the HLA class II peptide ligandome repertoire does not change drastically with expanded sampling from more proteins.

### C-Terminal Extended CLIP-Core Peptides Are Overrepresented at 40 °C

**Fig. 7:**
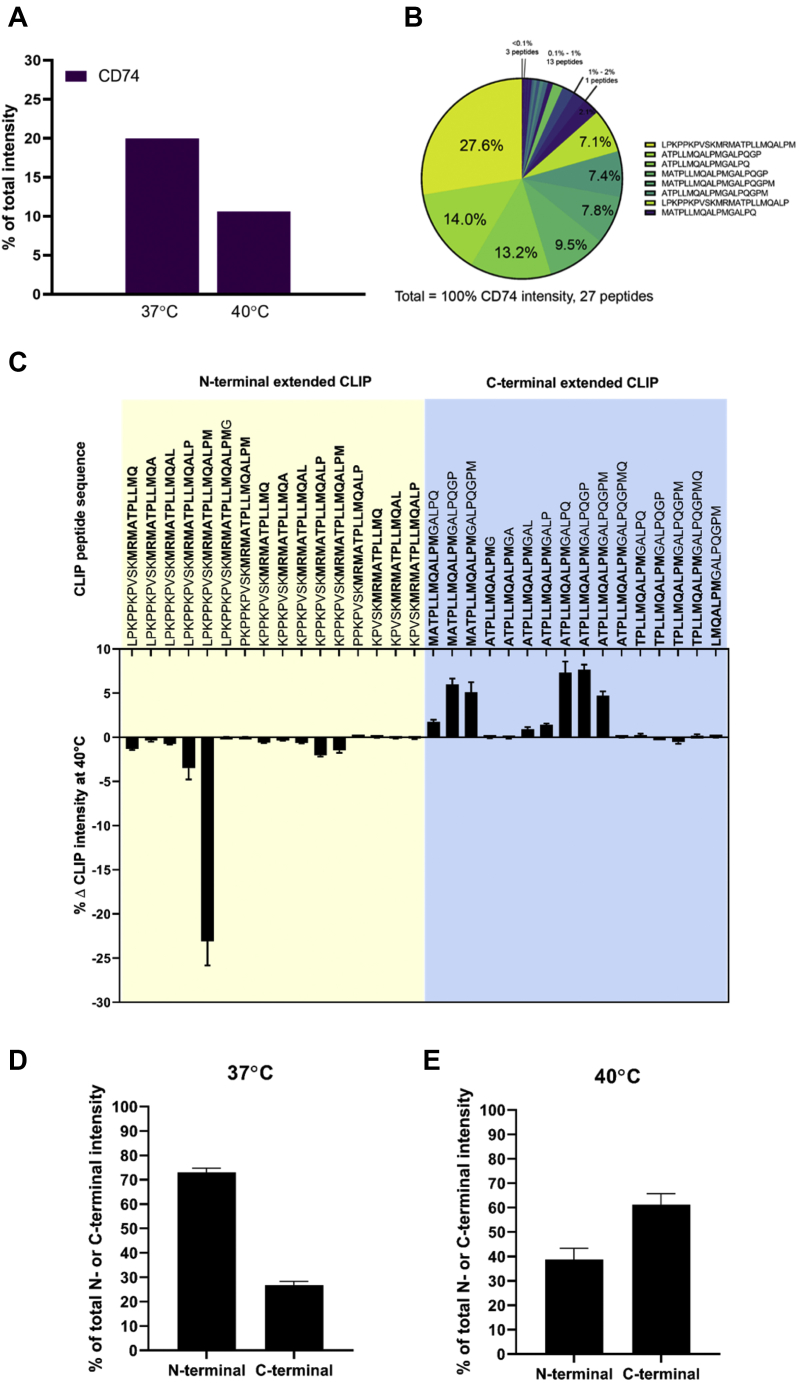
**Temperature induced changes in the HLA bound CLIP peptide repertoire.***A*, contribution of CD74 CLIP peptides to the total HLA class II ligandome at 37 °C and 40 °C. *B*, pie chart of all identified CLIP peptides at 40 °C, with the contributing intensity as a fraction to the total intensity of all CD74 peptides. *C*, percentage change in CLIP intensity at 40 °C *versus* 37 °C. *D*, percentage of N-terminal and C-terminal CLIP intensity at 37 °C. *E*, percentage of N-terminal and C-terminal CLIP intensity at 37 °C. HLA, human leukocyte antigen.

As shown earlier, 34 different laddered CLIP peptides were detectable in the HLA class II peptidome of cells cultured at 37 °C (Fig. 2*B*), making CD74 one of the most heavily sampled self-proteins in JY cells. Intriguingly, high temperature induced shifts in sampling from CD74, both in terms of individual CLIP peptide intensity, as well as the composition of the CLIP repertoire. As shown in Figure 7*A*, the total occupancy of CLIP on the HLA class II molecules decreased from 20% to 10% on exposure to high temperature. The most abundant species out of the CLIP repertoire at 37 °C, LPKPPKPVSKMRMATPLLMQALPM (51%), remained the most abundant at 40 °C, but its total contribution to the CLIP repertoire was halved to 28% (Fig. 7*B*). In addition, the next six most abundant CLIP variants contributed instead 60% of the intensity to the CLIP cluster. As such, the CLIP repertoire at 40 °C was more balanced and consisted of multiple equally abundant variants (Fig. 7*B*) instead of the at 37 °C dominant LPKPPKPVSKMRMATPLLMQALPM peptide (Fig. 2*C*). Quantitatively, N terminal and C terminal extended CLIP peptides were also differentially presented by HLA class II proteins when cells were exposed to high temperature (Fig. 7, *C*–*E*). Collectively, these data outline both the qualitative and quantitative changes in the HLA class II associated CLIP peptide repertoire when JY cells are exposed to high temperature.

Taken together, the upregulation in HLA class II proteins (Fig. 5*A*) together with decreased total CLIP occupancy seems to suggest additional priming mechanisms in preparation to present peptides from exogenous pathogens. It is intriguing that elevated temperature alone is sufficient to trigger these changes. The peculiar skew toward C-terminal extension on the other hand seemed to imply that CLIP peptide trimming may be influenced by temperature.

## Discussion

In this study, we returned to some of the basics of in HLA peptide presentation to expand our understanding of the fundamental rules underlying this process. By analyzing the proteome and the HLA class I and class II peptide ligandomes from the same batch of JY cells, we could directly compare and contrast the antigen presentation *via* class I and class II mechanisms in the same biological system. This enabled over 10,000 HLA class I and more than 2100 HLA class II peptide ligands to be purified and identified from the same batch of JY material (Fig. 1). While the resting HLA class I ligandome consisted of diverse peptide fragments from many more intracellular proteins, the HLA class II ligandome is dominated by peptide ligands originating from only a dozen different source proteins, contributing ~70% to the total MS intensity. In concordance with a previous report (58), we also observed that the dominant peptides loaded on HLA class II molecules were derived from mostly membrane proteins and HLA class I proteins, as well as CD74, thereby reflecting the unique biochemical processing and loading paths specific to HLA class II presentation.

In the absence of invading pathogens, peptide fragments of CD74 dominate the HLA class II ligandome of resting JY cells. While the contribution of the CLIP peptide was estimated to be ~60% in previous reports (44, 59), we found this proportion to be much more modest, at about 20% in our experiment. We believe this discrepancy arises largely from the lack of instrument sensitivity, such that other low-intensity class II ligands were not detectable, leading to a skewed quantitative estimate of CLIP peptide contribution. Indeed, with significantly increased MS detection sensitivity, we now also discover a larger repertoire of CLIP peptides, characterized by sequence laddering, particularly with both N-terminal and C-terminal extensions in addition to the CLIP-core sequence (Fig. 2).This supports the notion that CLIP is a set of different sequences centered around a di-leucine motif (13, 60). Even though CLIP was classically thought to anchor in the open peptide groove of HLA class II *via* four residues, we found M107 among these four to be dispensable in half of the CLIP peptides detected from our model system. On the contrary, the di-leucine sequence is conserved in 31 out of 32 CLIP peptides, suggesting that this region may be way more critical for binding to HLA class II molecules and that these four additional anchor positions may only play a subsidiary role to assist in the loading of the CLIP peptide repertoire. The importance of these additional anchor sites may then also vary depending on the HLA class II allele and specific peptide loading groove. Indeed, in support of our model, CLIP peptide sliding and flipped docking have been sporadically reported (61).

By employing a pathogen-free and artificially induced high temperature treatment in culture, we mimicked a 3-day fever state (21) to probe changes to the HLA class I and class II antigen presentation systems in B cells. This experimental setup may seem simplistic but was put together with much thought to focus on identifying host adaptations at the proteome and antigen levels. Even in the absence of real invading exogenous pathogens, we observed that our model system became adapted in 3 days for B-cell activation and various B-cell specific functions (Figs. 3 and 4), suggesting that temperature alone could trigger the cells and partly prepare them to tackle infections by means of upregulation of HLA class II proteins. In addition, the absence of real invading exogenous pathogens allowed us to observe the spontaneous loss of CLIP peptide binding to HLA class II proteins, as opposed to these peptides being competed off the HLA class II molecules by peptides originating from pathogenic proteins. Such a spontaneous loss of CLIP peptide binding would not have been discernable in a pathogen challenged system. We thus believe, conceptually, this is a very interesting observation and physiologically intriguing adaptation in the immune system that we show here to be highly specific to HLA class II presentation.

More intriguingly, we also observed that the CLIP peptide repertoire loaded on HLA class II proteins can change in composition at elevated temperatures (Fig. 7*B*). In our opinion, this is unlikely because of global changes in the class II peptide processing pathways, because a very large proportion of the other HLA class II peptide ligands remain unchanged (Fig. 6*B*), the HLA class II peptide processing proteins remain largely unregulated (Fig. 5*A*), and the top ten source proteins also did not change substantially (Fig. 6*C*). In fact, among these top ten proteins, CD74, the precursor to the CLIP peptide repertoire is the only protein to become significantly less sampled at the higher temperature (Fig. 7*A*). By aligning these pieces of evidence, high temperature appears to specifically increase the expression of HLA class II proteins (Fig. 5*A*) and change the occupancy of specific CLIP peptides on HLA class II molecules. This is also consistent with the critical role of CLIP in protecting the peptide loading groove from premature antigen loading before HLA class II proteins reach the endosome (13, 60). As such, the release of CLIP peptides at high temperature may indeed signal a preparatory step to present peptides of exogenous pathogens. Moreover, not only is the collective occupancy of CLIP peptides on HLA class II proteins halved at 40 °C (from ~20% to ~10%), the distribution of CLIP variants within the CLIP repertoire was also altered, in favor of peptides with C-terminal extensions beyond the CLIP-core (Fig. 7*D*). Specifically, the proportion of C terminally extended CLIP peptides increased from 39% to 61% during high-temperature treatment. This may further hint at temperature-induced changes in HLA class II peptide trimming, which may be interesting to explore in the future.

To further examine the coherence of our CLIP repertoire against other HLA class II ligandomes, we also analyzed the ligands from three publicly available HLA class II data sets (PXD004894 (4); PXD020011 (62); PXD012308 (63)). PXD004894 contains HLA class II ligandomes of 21 different melanoma cell lines. PXD020011 contains immature and mature dendritic cell ligandomes from three different donors as well as the paired CD19+ B cells and CD4+ and CD8+ T cells. PXD012308 contains HLA class II ligand data from seven different B cells (also JY), one T-cell line, and 15 different meningioma cell lines. In all cases where CLIP peptides were documented, they were derived from N-terminal extended CD74 peptides, in agreement with our observation that these CLIP species dominate in normal temperature conditions.

Although still marginally when compared to work on HLA class I ligandomes, in recent years also quite a few mass spectrometry-based HLA class II peptide profiling studies have been reported (64, 65, 66, 67, 68, 69). This growing interest may be linked in view of the strong disease correlation. Nonetheless, the fundamental rules in HLA class II antigen presentation still remain much less clear than for HLA class I. The fever state is one of the most common physiological responses that accompany infections, which may be accompanied by changes in preference for the CLIP repertoire, as we show here. However, the current study is limited to a single commonly used B cell line grown *in vitro*. In addition, HLA-DO and HLA-DM proteins were also below detection in the JY cell line, which precluded further investigations into the mechanism and efficiency of CLIP peptide exchange in this cell line system. Future work should establish if these temperature adaptations are conserved in more cell lines, and look into if these HLA-II proteins loaded with C-terminal CLIP are still inserted in the cell surface, although the approach we take here requires a significant input of cell material, which may not be compatible with tissue-level profiling. More fundamentally, it would be interesting to identify the mechanism of shift in CLIP presentation, as such information may enable a specific boost to the presentation of foreign antigens to intensify the trigger of the host immune system. This may lead to quicker natural clearance of such disease-causing pathogens, without too much interference on the normal HLA class I and class II peptide ligandome.

## Data availability

The mass spectrometry proteomics and peptidomics data have been deposited to the ProteomeXchange Consortium *via* the PRIDE (70) partner repository with the data set identifier PXD022930 or DOI: 10.6019/PXD022930. The raw file to sample mapping is described in supplemental Table S3.

## Supplemental data

This article contains supplemental data.

## Conflict of interest

The authors declare no competing interests.
